# Larvæ of Files Ejected from the Stomach

**Published:** 1849-01

**Authors:** 


*Larvæ of Flies ejected from the Stomach.* Mr. Editor,—The two *creatures* enclosed in this little box were thrown from a man's stomach, four weeks since, in the act of vomiting. The man is represented to be of "middle age," of a hardy, robust constitution, and in his ordinary health. Although not an intemperate man, in the usual acception of the term, yet he occasionally drank a little spirit, and it was "immediately after drinking a tumbler of hot gin sling," that he was taken with nausea and vomiting, and threw up these *animals*, if animals they be. The have been kept during the four weeks in a dry pill-box made of pasteboard, and yet one of them is still *alive and kicking.* I have transferred them to another box, and protected them with a lock of moist cotton to secure them from harm on their way to Boston. They were presented me by Dr. Grant, of Ossipee, a member of our Legislature now in session in this town, who will be pleased to answer any inquiries respecting the case. If the little one should be as active when you obtain him as he is now, I think you will consider him, as I certainly do, a queer fish to be derived from such a source. Very respectfully,

*Concord, N. H., Dec. 4th*, 184. THO. CHADBOURNE.

Immediately on the receipt of the box containing the larvæ, one of which was alive, we called on Augustus A. Gould, M. D., the distinguished entomologist, for his opinion in regard to them—and he has kindly sent us the following note.—ED.

DEAR SIR,—The animals you left with me are larvæ of a large fly (*syrphus?*), which live in the water. They are familiarly called *rat-tailed worms*. The rings of the tail are constructed to push out, like the joints of a telescope, so as to reach the surface of the water, and thus accommodate themselves to different depths. Through this tube they draw in air, and the end is protected by a circlet of hairs to prevent foreign subtances from entering the tube. Respectfully,

*Boston, Dec. 6th*, 1848. AUGUSTUS A. GOULD.

*Bost. Med. and Sur. Jour.*

